# Role of Neurotrophins in Neuropathic Pain

**DOI:** 10.2174/157015911798376208

**Published:** 2011-12

**Authors:** Dario Siniscalco, Catia Giordano, Francesco Rossi, Sabatino Maione, Vito de Novellis

**Affiliations:** Department of Experimental Medicine, Division of Pharmacology “L. Donatelli”, Second University of Naples, Via S. Maria di Costantinopoli, 16 - 80138 Naples, Italy

**Keywords:** Neurotrophins, NGF, BDNF, NT-3, NT-4, neuropathic pain.

## Abstract

Neurotrophins (NTs) belong to a family of structurally and functionally related proteins, they are the subsets of neurotrophic factors. Neurotrophins are responsible for diverse actions in the developing peripheral and central nervous systems. They are important regulators of neuronal function, affecting neuronal survival and growth. They are able to regulate cell death and survival in development as well as in pathophysiologic states. NTs and their receptors are expressed in areas of the brain that undergo plasticity, indicating that they are able to modulate synaptic plasticity.

Recently, neurotrophins have been shown to play significant roles in the development and transmission of neuropathic pain. Neuropathic pain is initiated by a primary lesion or dysfunction in the nervous system. It has a huge impact on the quality of life. It is debilitating and often has an associated degree of depression that contributes to decreasing human well being. Neuropathic pain ranks at the first place for sanitary costs.

Neuropathic pain treatment is extremely difficult. Several molecular pathways are involved, making it a very complex disease. Excitatory or inhibitory pathways controlling neuropathic pain development show altered gene expression, caused by peripheral nerve injury. At present there are no valid treatments over time and neuropathic pain can be classified as an incurable disease.

Nowadays, pain research is directing towards new molecular methods. By targeting neurotrophin molecules it may be possible to provide better pain control than currently available.

## NEUROTROPHINS: OVERVIEW

The neurotrophins (NTs) nerve growth factor (NGF), brain-derived neurotrophic factor (BDNF), neurotrophin-3 (NT-3) and neurotrophin-4 (NT-4) belong to a family of structurally and functionally related proteins and are a subset of neurotrophic factors. NTs are responsible for diverse actions in the developing peripheral and central nervous systems [1]. Indeed, NTs, once released by target cells, regulate the type and the number of afferent synapses by promoting the survival of discrete neuronal sub populations [2]. They are the important regulators of neuronal functions, affecting neuronal survival and growth, regulating differentiation, influencing cell fate choices and regulating neurite morphology. They are also able to regulate cell death and survival in development as well as pathophysiologic states. NTs and their receptors are expressed in areas of the brain that undergo plasticity, indicating that they are able to modulate synaptic plasticity. Rapid NT-mediated responses, such as changes in synaptic activity, probably result partly from the activation of second messengers and/or kinases that in turn affects ion channel function, neuro-transmitter release, and/or synaptic structure. Slow NT-mediated responses, such as NT-induced differentiation, depend on new gene expression [3]. Mature NTs are homodimeric proteins derived by proteolytic cleavage of precursor proteins encoded by different genes. NTs interact with two categories of cell surface receptors mediating neurotrophin actions: the Trk family (TrkA, TrkB and TrkC) of high affinity tyrosine kinase receptors and the low-affinity p75 neurotrophin receptor (p75NTR). TrkA (also known as neurotrophic tyrosine kinase receptor, type 1 [NTRK1]) is the high-affinity receptor for NGF; while for BDNF and NT-4, it is TrkB; for NT-3, it is TrkC [4, 5]. All members of NT family are able to activate the p75 receptor. Through the activation of these receptors, NTs activate many intracellular signalling pathways. For example, NGF binds and dimerizes its receptor TrkA; dimerization activates the receptor's intrinsic tyrosine kinase. Activated TrkA autophosphorylates several tyrosine residues present in the receptor's cytoplasmic domain. The phosphotyrosines serve as docking sites for adapter proteins and kinases such as phospholipase Cγ (PLCγ), phosphatidylinositol-3′ kinase (PI3K), and the adapter protein src homology-2 containing (SHC) [6]. These molecules are able to trigger multiple kinase bio-chemical cascades that culminate in the phosphorylation and activation of several transcription factors (i.e. Ras/Raf, the Cdc42/Rac/RhoG protein family, mitogen- and extracellular-regulated kinase (MEK), mitogen-activated protein kinase (MAPK), extracellular-regulated kinase [ERK]), which in turn induce direct gene expression [7]. NGF and BDNF are able to activate cyclic AMP response element-binding protein (CREB) by a calcium/ calmodulin-dependent kinase IV (CaMKIV)-regulated pathway [8], suggesting that CREB could play a central role in mediating NT responses in neurons.

Several neurodegenerative diseases and psychiatric disorders, such as Alzheimer's disease, Parkinson's disease, depression and substance abuse are associated with NT dysregulation [9]. Recently, NTs have been shown to be involved in the neuronal mechanism underlying neuropathic pain development and transmission. Due to their important role in this syndrome, NTs could represent a neuropathic pain pharmacological target for the next future of pain-medicine.

## NEUROPATHIC PAIN: PHYSIOLOGY AND CLASSICAL PHARMACOLOGICAL TREATMENT

Neuropathic pain can occur secondarily to injury of the central nervous system, but it occurs most commonly in association with a primary lesion or dysfunction to the peripheral nervous system [10, 11]. These injuries can be iatrogenic (amputations, cholecistectomy, mastectomy, dental avulsion), or can be due to trauma, tumour compressing peripheral nerves, toxins used for chemotherapy, metabolic (diabetes) and viral diseases (Herpes Zoster), nerve compression and inflammation following disc herniation.

Neuropathic pain is often associated with a continuous burning condition, accompanied by abnormal sensory symptoms, such as hyperalgesia (an increased response to a stimulus which is normally painful; patients with hyperalgesia perceive pain spontaneously) and allodynia (pain as a result of a stimulus which does not provoke pain; patients with allodynia do not feel constant pain; indeed in the absence of a stimulus there is no pain) [12]. Central mechanisms controling such spread of pain, arise from neurochemical and functional changes, and therefore, neuropathic pain should be considered a neuropathological condition [13].

Dorsal horn spinal cord is the first CNS-area involved in the pain control and processing. Nociceptive and non-nociceptive afferent pathway converge in this site, is that the specific neurons transmit nociceptive information to higher centres in the brain. Moreover, nociceptive neurons located in the superficial Lamina I (marginal layer) and in the Lamina II (substantia gelatinosa) and receiving direct synaptic input from A-delta fibers, together with indirect input from C-fibers *via *neurons in Lamina II, project nociceptive signaling to higher brain centres [14].

Central anatomical and biochemical modifications follow peripheral nerve lesions: central sensitization, supra-spinal, spinal re-organization and changes in the inhibitory pathways. Primary afferent neurons and the sympathetic neuron sproutings are the most important peripheral modifications [15]. Neurotrophic factors and several cytokines, such as interleukin-1 (IL-1) and tumour necrosis factor-alpha (TNF-alpha), can be responsible for the sprouting formation [14, 16]. Other modifications are: nociceptor sensitization, alterations in ion channel expression, ectopic and spontaneous discharge [14]. This latter alteration is a massive increase in the level of normal firing, following nerve injury of the afferent neurons and it occurs close to the site of the injury. Probably, after the nerve injury, an altered gene expression of the voltage-gated sodium channels causes an increased firing and, in turn, sensitization of peripheral nociceptors [17]. Neurotrophin, in particular NGF, may participate in this altered gene expression [18].

Neuropathic pain is associated with hyperactivity of excitatory (glutamatergic) transmission and hypoactivity of the inhibitory (GABAergic) transmission. GABA and glutamate are the key neurotransmitters involved in the neuropathic pain pathways [19]. Glutamate released by C-fibers leads to an increasingly enhanced response of the dorsal horn neurons: this phenomenon of central sensitization is called “wind-up” [20]. As for GABA, it is known that increasing its neurotransmission in the brain insular cortex enhances the anti-nociceptive response. GABAergic neurons control the projections modulating the nociceptive responses: the noradrenergic bulbo-spinal projections from the insular cortex to the locus coeruleus and the projections from cortex to amygdala [21].

The neuropathic pain syndrome is also responsible for changes in DNA expression. After peripheral nerve damage, the apoptotic genes mRNA expression levels of the *bcl-2* cell death-associated family in the lumbar dorsal horn of the spinal cord of neuropathic rats are modified [13, 22].

Tricyclic antidepressant and/or anticonvulsant drugs are the current available treatments for neuropathic pain, whatever its origin. All these treatments, however, even when well used, provide a long lasting relief only in a limited percentage of patients (30%, e.g. comparable to placebo), before pain reappearing. Classical pharmacological treatment of neuropathic pain includes: lidocaine, lamotrigine, acetaminophen, dextromethorphan, carbamazepine, gabapentin, valproic acid, opioid analgesics and tramadol hydrochloride. Only 40-60% of patients achieve partial relief [23]. The diversity of therapeutic approaches sharing an equal percentage of failure suggests that each of them targets only a few of the multiple pathological changes observed during the development of the disease. The overall approach to neuropathic pain is partial and palliative, i.e. it targets the epiphenomenon but not the cause.

## NEUROTROPHINS IN NEUROPATHIC PAIN

### Nerve Growth Factor

Increased emerging studies, highlighted key roles of NGF in neuropathic pain conditions [24-29], although originally discovered as a trophic factor for sympathetic and sensory neurons during its development. 

NGF acts as a pathogenic pain mediator [30, 31]. Its levels are elevated in several painful conditions and its administration in rats or for that matter in humans results in pronounced mechanical and thermal hyperalgesia [32, 33]. 

The increase in the level of normal firing in the afferent neurons after nerve injury is due to an altered expression of several types of sodium channels, such as the voltage-gated sodium channels [17]. The mechanisms responsible for the changes in the channel expression are not yet known, but NT supply can be involved [14]. Indeed, NGF is able to up-regulate the voltage gated sodium channels expression in neuropathic pain states, that it is critically linked to sensitization of peripheral nociceptors [34].

More recently, in the chronic constriction injury (CCI) model of peripheral neuropathy in rat, it has been demonstrated that NGF was increased in the ipsilateral dorsal root ganglia (DRG), in the spinal cord and in the periaqueductal grey matter (PAG). Systematic treatment with the anti-hyperalgesic and neuroregenerative compound acetyl-l-carnitine (ALCAR) was able to normalize NGF levels [35]. In addition, the NGF expression is increased in the red nucleus of the brain of neuropathic rats [36], as well as in DRG of rat pups during postnatal life after complete Freund's adjuvant (CFA)-induced peripheral inflammation [37].

One of the proposed mechanisms of action of NGF might through up-regulated several pain-related genes in the primary sensory neurons of DRG. Indeed, genes codifying for substance P, calcitonin gene-related peptide, TRPV1, Na(v)1.8 and Na(v)1.9 sodium channels and mu opioid receptor (MOR) are up-regulated by NGF [38-41].

In addition, the pain-activated glia are important sources of NGF [42]. In the neuropathic pain model obtained by peripheral axotomy, the initial NGF levels decline, due to disruption of transport from target tissues. NGF levels then rebound, as satellite glial cells begin to synthesize NGF and supply it to the neurons. Indeed, NGF and NT-3 synthesis are up-regulated in the satellite cells surrounding neurons in lesioned DRG as early as 48h after a nerve injury, indicating that the satellite cell-derived NTs are involved in the induction of sympathetic sprouting following peripheral nerve injury [43].

NGF plays a key role also in post-operative pain. Surgical trauma induce changes in the central nervous system pain modulating mechanisms. A Post-operative pain can trigger the central sensitization of the spinal cord and, in turn, can develop into a chronic neuropathic pain. NGF is released in incised tissue and contributes to hyperalgesia in incisional pain. NGF mRNA is increased and the large-molecular-weight form of NGF protein is expressed in the region adjacent to the incision at the plantar aspect of hind paw in rat [44].

Recent research is moving to target NGF-mediated pain. Interesting, drugs blocking NGF are proving to be effective in animal models of pain in which the non-steroid anti-inflammatory drugs (NSAIDs) and opiates have no effect in pain relief [45]. The development of humanized monoclonal antibodies to NGF or its TrkA receptor, and the sequestration of NGF using TrkA domain 5 (TrkAd5), a soluble receptor protein that binds NGF with picomolar affinity seem to be effective in a number of preclinical models of pain [46]. Targeting either the extracellular NGF binding domain of TrkA or probably its intracellular tyrosine kinase domain with small-molecule TrkA antagonists that can be the next step in the antibody-based therapy. In fact, the anti-NGF treatment was able to reduce neuropeptide levels and the nociceptive sensitization in rats with complex regional pain syndrome type I. According to the authors, anti-NGF antibodies prevented mechanical nociceptive sensitization, reduced spinal cord dorsal horn Fos expression and the sciatic nerve neuropeptide content [47]. The anti-NGF antibodies used in this study were the TrkA-immunoglobulin G (TrkA-IGG) fusion proteins able to bind NGF, thus blocking the binding of NGF to the TrkA and p75-NGF receptors and inhibiting TrkA autophosphorylation [48]. Anti-NGF antibodies were able to reverse the tactile allodynia and thermal hyperalgesia in the complete Freund's adjuvant-induced hind-paw inflammation, spinal nerve ligation, chronic constriction injury and streptozotocin-induced neuropathic pain models in rats and mice [28]. In addition, TrkA receptor also represents a suitable target for the antibody-based drugs. The anti-TrkA monoclonal antibody MNAC13 has been shown to posses a significant anti-allodynic effect on neuropathic pain, inducing functional recovery in mice subjected to sciatic nerve ligation [49]. Interestingly, the molecular strategies directed to blocking Trka-mediated events are showing promising results. An intrathecal administration a of antisense oligodeoxynucleotides to TrkA was able to decrease burn-induced primary mechanical hyperalgesia in dose-related manner in the rat [50]. A non-peptidic molecule, ALE-0540, inhibits the binding of NGF to TrkA, and, of course, signal transduction and biological responses mediated by TrkA receptors. Administration of ALE-0540 in rats decreased allodynia in the L5/L6 ligation model of neuropathic pain [51]. Given that studies, NGF blockers could be a new option for the next generation in neuropathic pain drugs.

On the other hand, NGF may produce beneficial effects in neuropathic pain. It has been demonstrated that NGF could be effective in restoring homeostatic conditions in the spinal cord and maintaining analgesia in neuropathic pain animals. Intrathecal NGF administration reduced allodynia, thermal hyperalgesia, reversed neuro-glial morphological and the molecular changes occurring in neuropathic animals. Moreover, as an NGF-mimetic peptide was shown to reduce neuropathic behaviour and restore neuronal function in a rat model of peripheral neuropathic pain [52, 53]. In a phase-II trial, it has been found a positive effect of recombinant human NGF on neuropathic pain in HIV-associated sensory neuropathy, even if injection site hyperalgesia was frequent [54].

Taken together these studies suggest that NGF is once of the major mediators of neuropathic pain, indicating a new therapeutic target for pain relief.

## BRAIN-DERIVED NEUROTROPHIC FACTOR

BDNF shows similar hyperalgesic effects to NGF [35]. BDNF is involved in the central sensitization and synaptic plasticity in the spinal cord. It has been shown to contribute to the development and maintenance of neuropathic pain by activation of the dorsal horn NR2B-containing NMDA (NMDA-2B) receptors [55]. Indeed, in the spinal nerve ligation (SNL) model of neuropathic pain, BDNF expression is significantly up-regulated in the spinal dorsal horn in SNL rats. The maximal enhancement of BDNF expression occurred in an early stage (24-48h) after SNL, indicating that BDNF/TrkB-mediated signalling pathway within the spinal cord could be involved in the induction of neuropathic pain in early stage after nerve injury. BDNF expression is also significantly up-regulated in DRG sensory neurons in lumbar 5 ventral root transection model of neuropathic pain [56]. These data highlight that an increased BDNF expression in DRG primary sensory neurons and spinal cord dorsal horn can be an important factor in the induction of neuropathic pain. The primary sensory neurons synthesized BDNF that can be anterogradely transported to the central terminals of the primary afferents in the spinal dorsal horn, enhanced its local expression [26]. 

It has been demonstrated that the oral administration of protein kinase inhibitor, protein phosphatase 1 (1NM-PP1), is at doses that blocked phosphorylation of TrkB in the spinal cord and is able to prevent the development of tissue- or nerve injury-induced heat and the mechanical hypersensitivity in mice, indicating that TrkB signalling is not only an important contributor to the induction of heat and mechanical hypersensitivity produced by tissue or nerve injury but also to the development and persistence of neuropathic pain [57, 58].

BDNF is strongly involved in the axonal sprouting of intraspinal serotonergic fibers following the dorsal root injuries (DRIs). This model of neuropathic pain results in the permanent disconnection of nerve roots from the spinal cord and leads to the sensory impairments, loss of sensation and axonal sprouting of intraspinal serotonergic fibers. Endogenous BDNF is required for this sprouting of serotonergic axons. Its upregulation is mediated by activated microglia [59]. Besides serotoninergic sprouting, peripheral nerve injury also results in sprouting of the spinal noradrenergic fibers in rodent dorsal horn lumbar spinal cord [60]. BDNF modulates the noradrenergic system; indeed, spinal nor-adrenergic fibers were found increased in L4-L6 DRG ipsilateral to injury and in lumbar spinal cord following nerve injury. After intrathecal infusion of BDNF antiserum spinal noradrenergic sprouting was prevented. Authors suggest that increased BDNF synthesis and release drive the spinal noradrenergic sprouting following nerve injury, and that this sprouting may paradoxically increase the capacity for analgesia in neuropathic pain [60].

One of the proposed mechanisms of action of BDNF in neuropathic pain can be through enhanced neuronal sensitivity to painful stimuli and an increased co-expression of thermo-TRP channels. Indeed, it has been demonstrated that BDNF is able to regulate the pattern of expression and the level of activity of the transducer channel TRPV1 [61]. A well-known receptor that has been implicated in the mechanical, chemical and thermal nociceptive stimuli transmission. It is noteworthy that TRPV1 is also regulated by NGF [62]. Another possible mechanism concerns the involvement of microglia in the pain development. In fact, in response to the peripheral nerve injury, the spinal cord activated microglia P2X(4) receptors (P2X(4)R) are over-expressed. Activated P2X(4)R leads to the release of BDNF from microglia [63, 64].

As for NGF, an antibody-based therapy is showing good results for targeting BDNF. Indeed, anti-BDNF antibodies were able to postpone the mechanical hyper-nociception in mice with brachial plexus avulsion pain model [65]. A repeated intrathecal pre-treatment with specific anti-BDNF antibodies was able to abolish thermal hyperalgesia induced by nerve ligation in mice. Additionally, the thermal hyperalgesia was completely suppressed by a repeated intrathecal injection of specific antibody to its full-length TrkB [58].

The BDNF also has another side. Indeed, recombinant adeno-associated viral vector-mediated over-expression of BDNF has reversed the pain-like behaviours in neuropathic rats, suggesting that by changing the levels of neurotrophins in the spinal cord micro-environment following nerve injury, it is possible to recover normal function [66].

## NEUROTROPHIN-3

Neurotrophin-3 has been shown to have antagonistic effects to NGF in the pain processing, through negative modulation of NGF receptor expression and associated nociceptive phenotype in intacted neurons [14]. It has been demonstrated that NT-3 reduces an over-expression of Na(v)1.8 and Na(v)1.9 channels in DRG neurons of the neuropathic rats [34]. As mentioned above, the NT-3 is able to interact with the TrkC receptor, but it also possesses the ability to signal *via *a TrkA receptor. NT-3 could act on Na(v)1.8 and Na(v)1.9 channel of expression. Interestingly, NT-3 is able to cause tyrosine-phosphorylation of the glial cell line-derived neurotrophic factor (GDNF)/Ret receptor and activation of PI3-kinase/Akt pathway [67]. In addition, NT-3 is able to reverse the CCI-induced thermal hyperalgesia through a down-regulation of TRPV1 receptor expression [68].

Moreover, the NT-3 is able to downregulate the potassium Kv channel of gene expression in DRG neurons following the nerve injury [69]. 

The role played by NT-3 in neuropathic pain is still less clear. It has been proposed that NT-3 can be involved in a long-term change of neuronal excitability. Indeed, it has been proved that NT-3 to promotes an extensive growth of lesioned axons in rat crushed dorsal columns [70].

Neurotrophic factor delivered by an adenovirus-based gene therapy can be a promising strategy for the prevention of neuropathic pain [71]. Recombinant adenovirus encoding NT-3 intramuscular injected in rats with streptozotocin-induced diabetes has decreased the denervation observed in this model of diabetic neuropathy [72]. 

Taken together these findings are consistent with an analgesic role for NT-3. Conversely, the NT-3 other side: intrathecal administration of NT-3 antisense oligonucleotides attenuates nerve injury-induced sprouting and allodynia [73].

## NEUROTROPHIN-4

It is not yet clear the role played by NT-4 in neuropathic pain physiology. NT-4 is synthesized by DRG and expressed in the rat spinal cord [58]. It is a ligand of the TrkB tyrosine kinase receptor, but it mediates to diverse effects in relation to BDNF [74]. It has also been demonstrated that repeated injections of a specific antibody to NT-4 failed to reverse the thermal hyperalgesia caused by sciatic nerve ligation in mice [58]. These results indicate that NT-4 can be an essential component of nociceptive processing. However, its use in the pharmacological treatment can be suitable. Indeed, the addition of NT-4 to injured nerves improves their regeneration potential and can affect axon guidance [75]. In addition, NT-4 drives peripheral nerve regeneration through regulation of the expression of myelin-associated glycoprotein, myelins basic protein, and low-molecular-weight neurofilament protein [76]. However, further studies are needed to better elucidate the role of NT-4 in a neuropathic pain.

## CONCLUSIONS

Neuropathic pain is a very complex disease, involving several molecular pathways. Due to its individual character, the treatment is extremely difficult. The current available drugs have a generalized nature and act only on the temporal pain symptoms rather than being targeted towards the several mechanisms underlying the generation and propagation of pain. Despite over fifty years of research there been no valid treatments over time and the neuropathic pain can be classified as an incurable disease without treatment. The increasing number of negative clinical trials of pharmacological treatments for neuropathic pain highlights the need for new molecular targets. NTs represent new promising potential targets for the next-future drugs for neuropathic pain relief. 
